# Preclerkship Medical Students’ Use of Third-Party Learning Resources

**DOI:** 10.1001/jamanetworkopen.2023.45971

**Published:** 2023-12-04

**Authors:** Emily C. N. Lawrence, C. Jessica Dine, Jennifer R. Kogan

**Affiliations:** 1 Michigan Medicine, University of Michigan, Ann Arbor; 2 Perelman School of Medicine, University of Pennsylvania, Philadelphia

## Abstract

**Importance:**

Undergraduate medical education increasingly relies on asynchronous, virtual learning; and medical educators have observed students engaging in self-directed learning outside of their institutional curriculum using widely available third-party resources. If medical educators better understand how students are learning, they may uncover novel opportunities to improve preclerkship education.

**Objective:**

To explore how and why preclerkship medical students use third-party learning resources.

**Design, Setting, and Participants:**

This qualitative study recruited second-year medical students from 7 public and private allopathic US medical schools and conducted 7 virtual focus groups (1 per institution) from September 2022 to January 2023, exploring how and why students use third-party resources. Data were iteratively analyzed in parallel with focus groups using constructivist grounded theory methodology. Data analysis was performed from October 2022 to February 2023.

**Results:**

Fifty-eight second-year US medical students who had used a third-party resource at least once participated; 36 (61%) identified as women; 13 (23%) identified as Asian, 6 (11%) as Black, 30 (53%) as White, 6 (11%) as multiracial, and 4 (7%) as other; 6 (10%) identified as Hispanic, Latino, or Spanish origin, and 52 (90%) identified as non–Hispanic, Latino, or Spanish origin; 48 (83%) were aged 23 to 25 years. Participants described engaging in a cyclical process of deciding whether and how to use third-party resources. Four broad themes were identified: (1) hearing about resources, (2) selecting resources, (3) using resources, and (4) tensions and possible solutions. Participants largely heard about third-party resources from peers and turned to resources out of dissatisfaction with some aspect of their medical school curriculum. Students used resources in various ways that were user-dependent and context-dependent. Participants endorsed multiple benefits over their in-house curricula, particularly efficiency, clarity, and concision. Tensions included navigating resource drawbacks and the perception of an antagonistic relationship between medical schools and third-party resources. Participants suggested that medical schools examine the resources, recommend specific ones, integrate them into the curriculum, and subsidize their cost.

**Conclusions and Relevance:**

In this qualitative study of preclerkship medical student use of third-party resources, participants perceived that the resources had numerous benefits for learning and suggested that medical schools should more formally acknowledge and integrate their use.

## Key Points

**Question:**

How and why do medical students use third-party medical education resources?

**Findings:**

This qualitative study of 58 second-year medical students found that participants used resources in various ways and described multiple benefits compared with in-house curricula, particularly efficiency, clarity, and concision; tensions included navigating resource drawbacks and the perception of an antagonistic relationship between medical schools and third-party resources. Participants suggested that medical schools examine the resources, recommend specific ones, integrate them into the curriculum, and subsidize their cost.

**Meaning:**

These results suggest that there are opportunities for medical educators to incorporate third-party resources into the curriculum or learn from their strengths as perceived by students.

## Teaser

This qualitative study explores how and why medical students use third-party educational resources outside of their institution’s curriculum.

## Introduction

Undergraduate medical education is transforming, with increased reliance on asynchronous, virtual learning^1^ as medical students are assuming more control of their preclerkship education.^2^ One factor enabling this is the widespread use of third-party resources (ie, learning resource not created by the medical school) and the decreased reliance on the medical school curriculum. Although medical students’ reliance on third-party resources is well documented,^3^ there remains limited understanding of why or how students use them.

The 2022 Association of American Medical Colleges (AAMC) Year 2 survey reported that approximately 70% of students used non–institution-created online videos (eg, YouTube) and other online content (eg, Wikipedia) daily or weekly for medical education information.^3^ The same study reported that only 41.5% of surveyed students attended in-person preclerkship courses “most of the time” or “often.”^3^ A single-institution survey of second-year students found that a greater percentage used commercially available online lectures compared with institutional lectures (84% vs 62%).^2^ Another single-institution survey found that few students reviewed formal coursework to prepare for United States Medical Licensing Examining (USMLE) Step 1 and instead relied on third-party resources.^4^ A 2-institution quantitative study comparing student preferences for medical school vs third-party resources found participants preferred third-party lecture length, teacher effectiveness, and material quality.^5^

There is a notable research gap concerning students’ decision-making processes about when and how to use third-party resources within the context of their school curriculum. If medical educators better understand the students’ approach to learning, they may uncover novel opportunities to improve preclerkship education. Therefore, the purpose of this study was to explore why and how medical students use third-party resources. Unlike prior survey-based studies, we used a constructivist approach, drawing on the principles of self-directed learning theory, to investigate the usage patterns and motivations behind medical students’ engagement with third-party resources. A constructivist approach aims to understand and construct meaning from participants’ perspectives, emphasizing the cocreation of knowledge between researchers and participants through iterative data collection and analysis.

## Methods

This qualitative study was deemed exempt by the University of Pennsylvania institutional review board as it posed little to no risk to participants. Participants provided informed written consent. We followed the Standards for Reporting Qualitative Research (SRQR) reporting guideline.^6^

### Design

We conducted focus groups using grounded theory methodology. Our analysis was informed by our sociocultural worldview and our roles at the time of writing. One of us (E.L.) was a fourth-year medical student. Two of us (J.K. and J.D.) have extensive experience in medical education curriculum development and assessment. Our perspectives informed the study design, implementation, and interpretation of results.

### Participants

In the fall of 2022, we recruited groups of second-year, predominantly preclerkship students from a convenience sample of 7 US geographically diverse private and public allopathic medical schools. These institutions have variable preclerkship curriculum structures (eg, traditional lectures, flipped classrooms, problem-based learning) and approaches to assessment. We partnered with leaders at each school to obtain institutional approval to share study information with or recruit participants by email. Eligible participants had to have used a third-party resource at least once. We accepted students until each institution’s focus group reached 10 participants with a goal of 5 to 10 participants per group. Participants received a monetary incentive of $50.

### Data Collection

Participants completed an online demographic survey prior to the focus group. Demographic characteristics, including race and ethnicity, were assessed to compare focus group composition with US medical student demographics as assessed by the AAMC Year 2 Questionnaire. We developed a focus group script asking about use of third-party resources in relation to use of school curricula. We asked participants to identify and discuss their reasons for use or nonuse, resource benefits and drawbacks, and what they wished their school knew about the resources (eAppendix in Supplement 1). Focus groups explored participants’ use of specific resources on the market, including but not limited to Pathoma, Sketchy Medical, Boards and Beyond, UWorld, AMBOSS, First Aid, and crowdsourced free materials such as the Zanki and Anking spaced-repetition flashcard decks. We conducted a pilot focus group with 6 fourth-year medical students at our institution after which we made small revisions to the focus group script. We revised the script as we conducted focus groups concordant with grounded theory technique.^5^ We ensured data saturation by using probing questions, exploring alternative views and perspectives, and member checking during the focus groups. Focus groups were held until no new themes were identified.

One study author (E.L) facilitated all focus groups via video conference (Zoom Video Communications) between September 2022 and January 2023. Each single institution focus group lasted 70 to 90 minutes. We recorded and transcribed focus groups using web-based Trint automated transcription software (Trint Ltd). One author (E.L) validated and deidentified transcriptions.

### Data Analysis

We iteratively analyzed data using constructivist grounded theory methods, including initial open coding, focused coding, and memo writing (NVivo software version 1.7.1 [QSR International]).^7^ We continued to analyze the data through discussion, engaging in constant comparison between themes and examination of associations between themes and new data as they were collected.^7^ We shared results with a sample of participants to ensure accuracy of interpretation. Data were analyzed from October 2022 to February 2023.

## Results

Among 58 medical students who participated in this study, 36 (61%) identified as women; 13 (23%) identified as Asian, 6 (11%) as Black, 30 (53%) as White, 6 (11%) as multiracial, and 4 (7%) as other; 6 (10%) identified as Hispanic, Latino, or Spanish origin, and 52 (90%) identified as non–Hispanic, Latino, or Spanish origin; 48 (83%) were aged 23 to 25 years. Focus groups each had 5 to 10 participants (Table 1).

**Table 1.** Demographic Characteristics of Participants at 7 Medical Schools, 2022-2023^a^

Characteristic	Participants, No. (%) (N = 58)
Gender	
Men	22 (39)
Women	36 (61)
Nonbinary	0
Race^b^	
Asian	13 (23)
Black	6 (11)
White	30 (53)
Multiracial	6 (11)
Other^c^	4 (7)
Ethnicity	
Hispanic, Latino, or Spanish origin	6 (10)
Non-Hispanic, Latino, or Spanish origin	52 (90)
Age, y	
20-22	3 (5)
23-25	48 (83)
26-27	5 (9)
≥28	2 (3)
Parents’ combined annual gross income regardless of dependency status	
<$25 000	3 (5)
$25 000-$49 999	1 (2)
$50 000-$74 999	1 (2)
$75 000-$99 999	4 (7)
$100 000-$149 999	12 (21)
$150 000-$199 999	11 (19)
$200 000-$249 999	4 (7)
$250 000-$299 999	5 (9)
$300 000-$399 999	4 (7)
$400 000-$499 999	0
$500 000 or more	3 (5)
Do not know or prefer not to answer	10 (18)

^a^ Participating schools: Perelman School of Medicine; University of Utah School of Medicine; Albert Einstein College of Medicine; Pritzker School of Medicine; Wake Forest University School of Medicine; University of Colorado School of Medicine; Zucker School of Medicine at Hofstra/Northwell.

^b^ Percentages may not total 100 due to rounding. In the case of race, percentages total greater than 100 due to individuals identifying as multiple races.

^c^ The other race category contained no specific race categories; it was an option available if participants wished to self-identify as other race not listed.

Participants described engaging in a cyclical process of hearing about third-party resources, considering multiple factors, and deciding whether to use the resource in question. When participants transitioned to a new block or module, they reentered the decision-making cycle, reexamined the resources they used, and often shifted their practices. We organized our results by the 3 cycle steps: (1) hearing about, (2) selecting, and (3) using resources (Figure). We described the tensions associated with this process and participant-recommended solutions.

**Figure.**
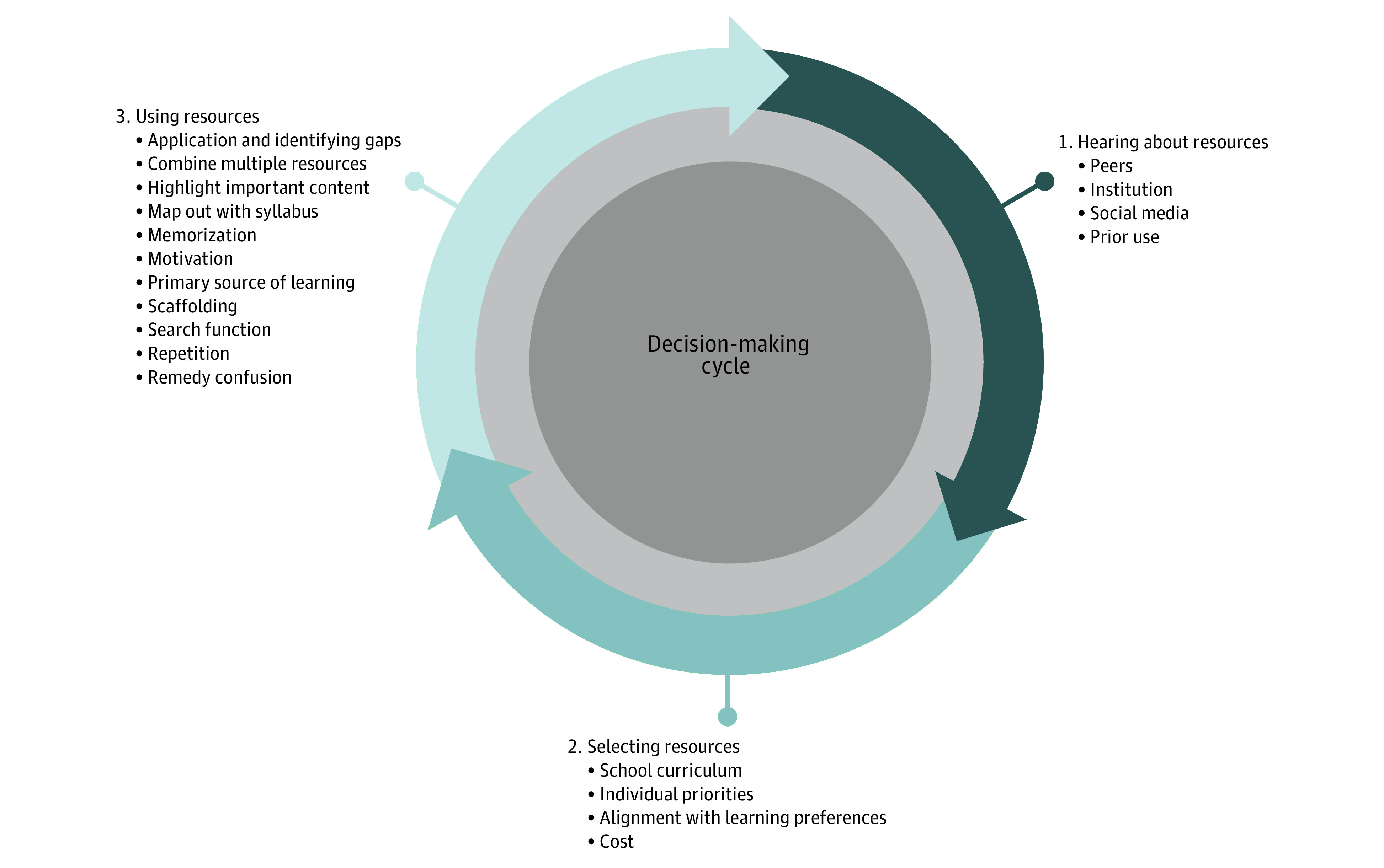
Decision-Making Cycle Participants described engaging in a cyclical decision-making process of (1) hearing about third-party resources, (2) selecting resources, and (3) using resources; and then reentering the cycle as the context of their learning changed.

### Hearing About Resources

Overwhelmingly, participants learned about third-party resources from peers, particularly more advanced students, at their own medical school but sometimes from friends at other schools. Knowledge transfer occurred through informal conversations and school-sponsored events. Knowledge of third-party resources became a form of social capital. Participants aimed to imitate high-performing students’ study strategies. “I think people identify people that they think do well in the class and try to emulate that....I think [spaced-repetition flashcard app] is 1 resource that has a lot of social pressure around it.” (Participant 3, focus group 6)

Less commonly, participants described learning about resources from social media or prior to medical school. Rarely did students learn about a resource from a course director or their school.

### Selecting Resources

In all focus groups, participants discussed the challenge and stress of navigating third-party resources given time and financial constraints. “I felt like I was missing something if I didn’t do it. It was stressful. It is overwhelming, the number of resources that you have to choose from.” (Participant 5, focus group 6)

Students relied on peer recommendations to select user-friendly, accurate resources but acknowledged some randomness and trial and error. Interestingly, participants rarely described questioning resource accuracy. Participants weighed many contextual factors when deciding if and how to use a third-party resource. We identified 4 factors that influenced resource selection: the school curriculum, course or module; student’s individual priorities; alignment with learning preferences; and cost. We present the factors in the order most commonly described (Table 2).

**Table 2.** Factors Considered Prior to Resource Selection

Factor	Example quote
School curriculum	“Lectures have a lot of extraneous information that is neither clinically relevant nor even covered on the test. I know some people have intellectual curiosity about experiments and things like that. I personally don’t. I find it a really annoying experience to listen to a lecture where someone’s going off on a ton of tangents or have classmates who are asking a bunch of tangential questions. It feels like a big waste of time when I could watch one of these videos in 10 minutes and get the core facts. And that is a real benefit to how well-organized these videos are.” (Participant 1, focus group 1)
	“[The school gave] a 2-hour lecture list of the names of all the bacteria or viruses you should know. They did not really go into depth very well. And it wasn’t an effective way to [learn]...You didn’t walk out knowing [the content]; you had to go watch some videos or read some text, do something to actually get it later. It honestly just made it time-efficient to [decide], I’m not going to go to class. I’m just going to watch these videos, and I’ll know the stuff I need to know.” (Participant 6, focus group 2)
	“We use very different resources depending on the block that we’re in. So, we keep accumulating resources as we go. This block that we’re currently in, we’re only using 3 out of the 5 or 6 resources that we already paid for.” (Participant 3, focus group 2)
	“Some of our systems tend to be taught by 1 lecturer and every lecture is given by that individual. And I find that it’s a lot clearer and I can focus on the lecture material…vs a course where each lecture was pretty much taught by a different doctor...a hodgepodge of information. I found it very unclear. For that, I had to watch every [online video resource] to understand that content because it didn’t make sense to me.” (Participant 6, focus group 3)
Individual priorities	“[Third-party resources] help me keep my sanity too. In college I burned myself out. In med school, I promised I wouldn’t do that. Discovering how to use the third-party resources helped me cut down time to not only do extracurriculars and research, but also have time for myself.” (Participant 10, focus group 3)
	“The thing that’s in the back of my mind is AOA, because I think you have to be top 15% of your class, which was motivating me to do better than just pass. In case I decide to go to competitive specialty, if anything can make me stand out is probably AOA in terms of grades.” (Participant 9, focus group 5)
Alignment with learning preferences	“The memory hooks really stick with me. I think there’s something about a cartoon that tricks me into thinking it’s a little bit more fun and engaging, especially when there are very long drug names that otherwise I don’t remember. It’s nice to have some kind of picture that’s weird and sticks with you, and I can remember the drug name so that when I’m in the clinical setting, I don’t feel clueless.” (Participant 5, focus group 1)
	“I personally learn well from videos, so I prefer the [online video lecture resource] style of learning. But [reference style resource] has been great because it has everything, and it highlights keywords. So even if you only know a little tidbit of something, you can type it in and generally you’ll get a result that puts it into the context that you’re looking for. And with the case-based curriculum, I think that it’s helpful to have the ability to click into basic science knowledge directly from an article about a disease pathology.” (Participant 7, focus group 7)
	“I did not use [spaced-repetition flashcard app] first year because it did not work for my brain, and it was very overwhelming at times. Just seeing everyone [use it] was stressful. I was like, ‘Shoot, I am totally doing this year wrong.’ But I just reached a point of fine, it’s not working for me.” (Participant 4, focus group 6)
	“I find [spaced-repetition flashcard app] incredibly boring. I really enjoy learning. And [spaced-repetition flashcard app] takes the joy out of learning for me a little bit because you’re drilling these little snippet facts into your brain. But as somebody who feels decently lost a lot of the time, it’s nice to have this tried-and-true study technique where I can’t go wrong.” (Participant 5, focus group 1)
Cost	“I didn’t want to fork out more money and I just don’t have the time or the bandwidth to use all of them to their potential. I decided to stick to the ones that I had rather than like get a bunch and not use them.” (Participant 1, focus group 2)
	“Most people who use [third party resource] have been using it a very long time. If you’re willing to commit that much time to it, then you can use it. But if you’re not willing to commit that much to it or you didn’t start early enough, it just isn’t that useful.” (Participant 1, focus group 7)
	“In terms of [reference style resource], I think paying for it was a barrier.” (Participant 4, focus group 7)

Abbreviation: AOA, Alpha Omega Alpha.

#### School Curriculum, Course, and/or Module

A key factor participants considered was whether the in-house curriculum met their needs. Participants expressed dissatisfaction with their schools’ clarity, organization, efficiency, and style of content presentation. Many participants desired more concise lectures, increased use of memory devices to facilitate retention, and opportunities to apply knowledge. Students selected third-party resources to meet these needs and address perceived curricular gaps. “I find [an online lecture resource] a lot more succinct. Our lecturers are so passionate, and I feel bad...but whenever they start talking about glaciers or water holes and I’m just super tired...which is why I like that little snippet: no intro, no conclusion. I don’t have to use extra time where I might zone out because I have a goldfish attention span.” (Participant 5, focus group 1)

Participants desired customized learning materials, often emphasizing the importance of alignment with USMLE Step examinations. “Sometimes lectures aren’t getting to the point of what’s important about a topic, maybe because it’s something [the lecturer is] super passionate about. They’re telling you all this interesting, cool stuff about a topic. But in the back of my mind, I’m like, is this going to be on Step? Is this going to be on my exam? I prefer a lecture that’s more straightforward and direct....We’ve had some lectures that [present] 20 years of research studies, but I don’t know if that’s what I have the capacity to remember.” (Participant 3, focus group 4)

Participants iteratively engage in the decision-making process, altering their use of resources for a new course or when their priorities shifted. “Every block, it ends up being a crapshoot at first to figure out which [resource] I like.” (Participant 6, focus group 7)

Frequently participants settled for what was good enough due to the costs (financial, time, energy) associated with trying resources. “Decision fatigue is a huge part of it. You could spend all your time trying to optimize it: this percent I should spend on this [resource], this is how much I should spend on lecture. Then there’s no time to be a person. Thinking about optimizing your studies is mentally draining. If you find stuff that works, sometimes that’s enough.” (Participant 4, focus group 3)

Participants shared that a pass-fail curriculum increased reliance on third-party resources. Even if resources did not align perfectly with school examinations, they were typically aligned well enough to pass. In contrast, pass-fail USMLE Step 1 scoring did not drive participants to rely on their school curriculum. “I know plenty of people who never look at the class stuff. They just do [third-party resources]; they pass every time. It’s probably the difference between 75 or 80 vs a high 90. The difference between remembering the 1 weird mutation on the slide or not, that you probably didn’t need to know anyway.” (Participant 10, focus group 3)

#### Individual Priorities

Participants preferred learning efficiently so they could pursue medicine-related (eg, research, shadowing, volunteering) or personal interests such as “exercising or going to the grocery store.” (Participant 4, focus group 5). Several participants described trying to distinguish themselves for their residency application while also maintaining wellness. “I’ve gotten more involved in things to differentiate myself in a world where Step 1 is pass/fail. I have this emphasis on efficiency....In an ideal world, I’d want to take the material from the third-party resources and from our faculty and try to get everything I can out of it. I have to make choices between learning certain material and keeping up with my research or with an extracurricular, I’m looking for the most efficient way to learn, which often seems to be these third-party resources.” (Participant 2, focus group 3)

#### Alignment With Learning Preferences

Participants selected resources that were enjoyable to use and aligned with their learning style or study preferences. There was significant variability in students’ preferred learning formats; a favorite approach for 1 student might be deemed ineffective by another. “There are different metrics of success…part of it is actually enjoying [the process]; you’re going to spend so much time studying in medical school, find something where it doesn’t suck all the joy out of learning.” (Participant 1, focus group 1) Occasionally, participants used a resource they did not enjoy if they perceived it offered a substantial benefit (ie, improved long-term retention).

#### Cost

Given many resources are subscription-based and “incredibly expensive” (Participant 3, focus group 4), participants considered cost, both financial and time-based, in their decision-making. Some resources required a substantial learning curve, particularly spaced-repetition flashcard apps. For some students, this learning curve was too great to surmount. Other resources required time to fully utilize, which was not always feasible. “I haven’t been able to stick with [the flashcard app] because it is so scheduled, and I was busy with other things, then coming back to a ton of cards and not being able to get through them. I pivoted to doing something else completely.” (Participant 4, focus group 1)

### Using Resources

Participants used resources in various ways which were, in part, dictated by the selected resource format (Table 3). Participants used resources to apply and identify knowledge gaps (ie, answering questions). Many participants used resources to identify what content was most important. Participants used resources with visuals or spaced repetition to assist with memorization. Frequently, participants used multiple resources simultaneously.

**Table 3.** How Preclerkship Medical Students Use Third-Party Resources

Use	Example quote
Application and identify knowledge gaps	“I really liked practice questions to poke holes or [determine] what can I not differentiate? What detail am I missing? Because for me that was way more effective.” (Participant 1, focus group 6)
Combine multiple resources	“I don’t watch lecture. If there’s a neuro block, I would go in the neuro section of the [spaced-repetition flashcard app crowdsourced] deck, and I would use whatever resource that I preferred, which usually ended up being a combination of [online video lecture resource] and [cartoon-style resource] in order to learn that content. And then afterwards, I’d go through the lecture titles, and if I saw the title of something that I haven’t learned or something that didn’t look familiar to me, then I would skim through the slides really quick and try to pick out any information that may be relevant on the exam that wasn’t covered by the third-party stuff.” (Participant 1, focus group 5)
Highlight most important content	“You could study medicine forever and never truly master it. The good thing about third-party resources is that they pound in high-yield material. Everything it’s presenting you with is effectively high yield. When you study based on that philosophy, you then absorb all the high-yield information, which ultimately, is the most important....I found that when I was studying primarily lecture first year, a lot of my studying time was spent reading through lecture and trying to pull out what is high yield and trying to figure out what I really need to know. I think the high-yield stuff should come first.” (Participant 4, focus group 5)
Map out with syllabus	“I scheduled all the [online video lecture resource] for the semester and matched them with what sounded like the right lecture…it helps me to know that what we’re going through in lecture actually is going to be relevant on Step and that it’ll mean something.” (Participant 7, focus group 2)
	“[There is] a document that has been shared by older students and it’s based on the lecture schedule and the third-party resources that go with that.” (Participant 9, focus group 6)
Memorization	“For me the biggest benefit of [cartoon-style resource] is it’s so easy to memorize large amounts of information. That’s why I found it so helpful for micro and drugs. I found that the only way is rote memorization, but then when you have the picture, I could recall it very easily even if it was a lot.” (Participant 1, focus group 1)
	“The first time I used [spaced-repetition flashcard app], I remember doing a question bank on that specific system, and I was really surprised by how well the information was sticking. I was like, wow, everything I know is because I’ve seen that card.” (Participant 5, focus group 3)
Motivation	“Seeing that little number [of scheduled spaced-repetition flashcard reviews], it’s like, hey, you haven’t done this. That is a very big motivator for me, as opposed to how I can always just push off lecture a day or two, but I know that [the flashcards] keep building up if I don’t do it daily.” (Participant 6, focus group 1)
Primary source of learning	“We had a lot of people in our class who would sit in the back of the classroom and watch [online video lecture resource] or watch external resources to get the information quickly or in a more efficient format than what they thought class would give them.” (Participant 2, focus group 6)
	“I didn’t even go to class for micro because I knew that [cartoon style resource] would help me.” (Participant 7, focus group 4)
	“My go-to is outside resources....I would like to almost entirely use outside resources because I think they have put so much work into them, and I know they’re good, and I know they have the material that I need to know.” (Participant 7, focus group 4)
Scaffolding	“I use [cartoon style resource] as a first pass. I’ll watch it the night before, the corresponding topics that we’re going to go over in class. And then when I go to class, I’m like, ‘Oh, like this was mentioned in the class and resource, so it’s probably important.’ I kind of use that as a way to determine what I think is going to be more important.” (Participant 2, focus group 4)
	“[My] goal is to watch the corresponding [video-based resource] prior to lecture. It helps prime me and provide a framework vs just jumping into lecture.” (Participant 2, focus group 5)
Search function	“If I have a very specific thing that I’m not understanding, I sometimes find it’s helpful to type in the name of the topic. (Participant 7, focus group 1)
	“Sometimes I’ll type a subject name in the [resource] search bar and it’ll pull up tailored questions to certain topics.” (Participant 5, focus group 2)
Repetition	“The data shows [spaced repetition resource] is the best for spaced repetition and long-term retention of the material. What I’m focusing on is long term retention of everything I’m learning currently and stuff we learned last year. I feel like that’s the best way to make sure you’re retaining everything you need to know.” (Participant 5, focus group 3)
Remedies confusion	“Any time I’ve used [video resource], it’s when something is not clicking. And the lecture was not helpful to me understanding it. I’m searching the concept to try to get...a succinct way of hearing another person explain it differently.” (Participant 7, focus group 1)
	“For topics that I found difficult and I just wanted like the big picture, I would use [resource].” (Participant 8, focus group 2)

Participants used the same resource differently. Many used resources to preview or scaffold content they would see in class (even when not required for flipped classroom sessions) whereas others used resources after class to remedy confusion. Some used resources during class time and others during interstitial time (eg, walking to school). Some used resources as their primary source of learning (at the extreme, entirely replacing the school curriculum) while others used resources as a supplement. Some described doing 2 full curricula (their school curriculum and third-party resources). Participants’ perceptions of the schools’ curriculum quality and efficiency influenced how resources were used.

### Tensions and Possible Solutions

Participants described 3 tensions related to third-party resources and how schools might address them. Here we describe these tensions and recommendations. Table 4 provides supporting quotes.

**Table 4.** Tensions and Solutions Navigating the Use of Third-Party Resources

Tension	Example quote describing tension	Example quote describing solution
Purpose of preclerkship education	I think a lot of [course directors] believe that their material is going to better prepare us to be better doctors. We all know that Step 1 is not the best test to determine if you’re going to be a good doctor. I think part of the curriculum is trying to cater toward the idea that medical school should prepare you to be a doctor and not just take a test. (Participant 5, focus group 5)	
	I completely stopped watching lectures this year. I felt guilty in the beginning because I [thought], this is disrespectful to our professors and there’s so much we can gain from their real-life knowledge. But then I told myself, we’re going to learn from clinicians on rotations. The purpose of preclinical is to learn basic science. It’s still a struggle in my head because I understand the utility of learning from doctors, but right now that’s not my focus. (Participant 5, focus group 3)	
Adversarial relation between school and resources	It makes you feel like these outside resources are illicit. Some professors explicitly relay how annoyed they are that nobody comes to lecture. I’d love to go to lecture if I felt that it was the best use of my time. When you have such limited time between school and outside obligations and just trying to stay on top of day-to-day life It’s a little frustrating when [the school] trivializes that and almost makes you feel bad about using [third-party resources] and tries to convince you that it’s not the right thing to do. (Participant 3, focus group 4)	I wish they wouldn’t see it as an either/or, [but rather as] giving students the tools that they need to succeed Not, oh, the school sucks and [this resource] is better. More like, given people are going to use this, how do we make it the most seamless for everybody and help people learn? (Participant 4, focus group 3)
	I’m using [third-party resources] instead of going to lecture, so I’m missing out on the opportunity to learn some of the more specific elements from the lecture, and I just feel bad that I’m not taking advantage of what I’m paying for, and now these instructors are teaching to an empty room. There’s some guilt there. There’s some feeling of missing out. But I keep doing it because the calculus makes sense for my individual learning. (Participant 2, focus group 3)	Many resources are used in a complementary way. I’m not sure professors or course directors view it that way. It almost feels slightly more antagonistic....I think having a more supportive approach, [such as] if you’re not an auditory learner and sitting through a 50-min lecture is not doing it for you, here are other ways to approach it. (Participant 1, focus group 1)
		I want to see the [Step] data on students who don’t go to lecture and only do third-party resources. I think the reason why [our school] has adopted so many is because they see it as helpful....Knowing those numbers is important so you can guide your students correctly. (Participant 9, focus group 5)
		It’s almost impossible to [make] every single [medical school] lecture better than the third-party resource lecture. If they can include a third-party resource into the [curriculum], that could be beneficial for every student. That way, we can use both at the same time, do more practice, or exclusively practice during class time instead of just doing in one hour not-so-efficiently what we’re already doing in 15 or 20 min at home. (Participant 3, focus group 2)
Concurrent cost of medical school and resources	My main complaint is we pay $40 000 to $80 000 a year, and I’m using 75% outside resources that I’m also paying for. I’ve often wondered if someone was given the opportunity to do self-study with outside resources, I bet they could pass Step 1 without paying huge loads of tuition. (Participant 1, focus group 2)	Ideally, the school recognizes that students are going to use these resources and creates a situation that is equitable, so that everyone can access them. (Participant 2, focus group 3)
	Paying tuition...that’s another reason why I want to watch the lectures and be as much a part of the [school] curriculum that I can, because it does seem absurd to be paying to go to medical school and only watching these videos that I could just watch at home online. (Participant 6, focus group 3)	

#### Purpose of Preclerkship Education

Participants expressed uncertainty about the primary purpose of preclerkship education and who should be the ultimate arbiter of preclerkship medical knowledge—their school or outside resources. Participants believed in-house curricula sufficiently prepared them for end-of-course examinations but questioned its adequacy for Step 1. Others expressed that while the preclerkship curriculum clinical content was interesting, the volume of information and the time required for their other endeavors necessitated focusing on the most important content, often defined as what USMLE tests. However, participants questioned whether what they were learning would help them become a “good doctor.”

#### Adversarial Relation Between the School and Resources

Participants perceived an adversarial relationship between their school and third-party resources. At some institutions, students were discouraged from using the resources while at others the negative regard was communicated more subtly. Participants believed they needed third-party resources to meet their educational and personal goals, but in doing so, felt guilty they were betraying their school. When participants substituted third-party resources for their school curriculum, they feared missing out on what their school could offer, such as face time with faculty, relationships with classmates, and information specific to their local community.

Participants wanted their schools to recognize the merits of third-party resources and create synergy with them, making it more feasible and straightforward to engage with and integrate both learning sources. Furthermore, participants wanted their schools to evaluate and endorse resources to reduce the amount of time and energy students spent trying out and identifying the best resources. Some participants suggested that schools use third-party resources for content delivery and restructure class time to focus on knowledge application and practice (eg, flipped classroom).

#### Concurrent Cost of Medical School and Resources

Participants commented on resource cost compared with medical school tuition given their belief that most, if not all, students were using third-party resources. Some participants attended school lectures to justify tuition costs. Participants wished their institutions would facilitate the subscription process, pay for or subsidize third-party resource costs.

## Discussion

This qualitative study explored medical students’ use of third-party learning resources. We found that participants use these resources to fill perceived needs in their medical education and that resources offer multiple benefits compared with in-house curricula. Participants engaged in an iterative and often frustrating decision-making process to select resources. They used resources in user-dependent and context-dependent ways while navigating substantial tensions. Identified themes were similar across schools and largely independent of a schools’ preclerkship curriculum structure.

Participants’ decision-making processes were inextricably linked to social context and time and financial constraints. Additionally, the school environment and US undergraduate medical education culture factored into decisions. The anxiety to perform well in medical school to be competitive for residency led participants to focus on materials that they perceived helped them achieve this goal. These patterns align with the concept of bounded rationality and fast-and-frugal heuristics.^8^ Bounded rationality is a decision-making model that balances the quality of a decision against the costs of extended deliberation. Fast-and-frugal heuristics are part of one’s “adaptive toolbox” and “employ a minimum of time, knowledge, and computation to make adaptive choices in real environments.”^9^ Medical students selected resources based on recommendations from more advanced students, particularly academically successful ones. Students used multiple heuristics to make choices, including the recognition heuristic (preference for the familiar), imitate-the-majority, and imitate-the-successful.^10^ While heuristics as mental shortcuts are subject to biases, they ease decisions when time is limited and information incomplete.^8^ Therefore, the use of these heuristics in medical students’ decision-making is reasonable and more feasible than attempting to collect every data point about each available resource. Many participants described engaging in satisficing, finding the good-enough option rather than attempting to arrive at the optimal decision.^11^ Even so, the selection process was difficult and burdensome, in part because of the large number of options and the high stakes involved, and students desired more guidance from their schools.

Participants’ use of third-party resources aligns with self-directed learning principles.^12^ If participants perceived in-house curriculum shortcomings, they identified third-party resources to meet their needs, frequently selecting resources based on their preferences for content presentation and the specific curricular shortcoming(s). Adult learners perform better and are more satisfied when they are empowered and self-directed in their learning.^12^ We found students prefer to have options for interacting with content, including video-based lectures, memory devices such as cartoons, spaced-repetition flashcards, and practice questions for self-assessment. The menu of options for content engagement offered by third-party resources enabled students to direct their learning, meet their individual needs for efficiency, gain and retain knowledge, and balance multiple competing interests. Third-party resources may help address the increasing calls to personalize medical student education.^13^ Our findings suggest there are opportunities for medical schools to enhance their preclerkship curriculum’s organization and concision, preferentially incorporating andragogical methods such as content application, memory devices, and spaced repetition.

Positioning third-party resources in opposition to the medical school curriculum generates a false dilemma for students. Participants’ resources use ranged from supplementing to replacing in-house curriculum. Many participants wanted to engage with both content sources but felt forced to choose given contextual limitations. The real or perceived adversarial relationship between medical school faculty and third-party resources was emotionally taxing for students, and the guilt some participants experienced using third-party resources surprised us. We recommend that schools explore and evaluate widely used third-party resources. There may be utility in incorporating high-quality third-party resources into or alongside the formal medical school curriculum to provide curricular options to students, enhance student learning, and reduce curricular burdens on institutions. If third-party resources are formally integrated into the curricula, schools should ensure that access, financial and otherwise, is available for all students, thereby mitigating inequities related to differences in students’ financial resources and social networks.^14^

We uncovered themes involving larger questions about the purpose of preclerkship education. While these questions have no clear answers, we find them worth asking. If the purpose of preclerkship education is, as some participants proposed, to develop basic science knowledge, we wonder how the role of medical schools might evolve. Some educators have heralded the “end of preclinical classroom instruction” and imagined a future in which “all preclinical preparation will be online, completed anywhere in the world by a multitude of learners from a handful of the world’s best professors.”^15^ This change would represent a seismic shift in medical education. However, we may already be moving toward such a reality, given our findings. While a narrower focus for preclerkship education may open new possibilities for access and equity, we wonder what might be lost. While several participants stated the ability to pass the USMLE Step 1 examination does not define a “good doctor,” the high-stakes examination loomed large, often defining for students what was deemed important in their preclerkship education. To mitigate these concerns, medical schools could focus on what they alone can offer preclerkship students: for example, professional identity formation, social determinants of health centering on the local community, mentorship, and research.

### Limitations

This study had limitations. First, this study did not examine whether third-party resources enhance learning. While participants described improved retention, further study regarding how resources affect learning is needed. Our findings may not be generalizable to other US medical schools, osteopathic schools, or schools outside the United States. However, participants’ demographics were similar to those reported by the AAMC.^3^ Given the study inclusion criteria required that students use at least 1 third-party resource, we could not explore perspectives of students who do not use these resources. Additionally, participants were first- and second-year medical students during the COVID-19 pandemic, potentially influencing their perceptions of and experience with third-party resources.

## Conclusions

In this qualitative study of preclerkship medical students’ use of third-party resources, we found that participants believed the resources had numerous benefits for learning and wanted schools to acknowledge and integrate their use more formally. These findings suggest opportunities to improve medical education for all students through personalization of learning and increasing equity and access to medical knowledge.

## Notes

Supplement 1. **eAppendix.** The Parallel Curriculum of Preclinical Medical Students: Focus Group Script V3

Supplement 2.
Data Sharing Statement
