# Supplementary material for: Preclerkship Medical Students’ Use of Third-Party Learning Resources
Source: JAMA Netw Open. 2023 Dec 4;6(12):e2345971. doi: 10.1001/jamanetworkopen.2023.45971 (PMC10696480; doi:10.1001/jamanetworkopen.2023.45971)
Supplement: Supplement 1. — eAppendix. The Parallel Curriculum of Preclinical Medical Students: Focus Group Script V3 [file jamanetwopen-e2345971-s001.pdf]

Supplemental Online Content

Lawrence EN, Dine CJ, Kogan JR. Preclerkship Medical Students' Use of Third-Party Learning Resources. *JAMA Netw Open.* 2023;6(12):e2345971.
doi:10.1001/jamanetworkopen.2023.45971

eAppendix. The Parallel Curriculum of Preclinical Medical Students: Focus Group Script V3

This supplemental material has been provided by the authors to give readers additional information about their work.

© 2023 Lawrence EN et al. *JAMA Network Open.*

eAppendix

The Parallel Curriculum of Preclinical Medical Students
Focus Group Script V3

Introduction
Good afternoon everyone. My name is Emily and I am here to lead the focus group for this study.

Thank you for participating in this study. We are going to spend the next 1.5 hours talking about your use of third-party study resources in medical school as well as the use of your home institution's curriculum. Just to be upfront, we have no role in the development of your institutional curricula, nor do we develop or work for any third-party resources, and so we hope you will feel comfortable being totally honest about your thoughts and perceptions. The overall study results compiled from all institutions will be shared with your institution, but not the specifics of our session today. All comments will be de-identified. The session is being audio-recorded so we ask that you speak clearly and slowly. The recording of the session will transcribed. Both the recording and transcription will be securely stored and password protected for your privacy.

I want to review a few ground rules for the focus group. It is important that we hear everyone's ideas and opinions, so we ask that only one person speaks at a time. Please remember that there are no right or wrong answers to questions- just ideas, experiences, and opinions which are all valuable. It is important that we hear all sides of an issue, both positive and negative. Next, please respect your fellow participants and do not share content from this discussion with others outside the group. Finally, as an aside, I am going to intentionally try to not insert my own opinions, experiences, and thoughts into the discussion so as not to influence the group. I want to mention that up front because I think sometimes it feels a bit awkward, but maybe that's just me!

Are there any questions? Let's take a moment and have everyone go around and introduce themselves.

Focus Group Questions

Now we will begin our discussion of what resources you use to learn in medical school and why. Let's start by taking a few moments to review the resources you shared in the pre-session survey. [Screen share the compiled list from the group, with additional tallies for each additional user beyond the first.]

- How did you first decide to start using third party resources?
- How did you go about choosing which resources to use?
    - What is the process of sorting through all of them and deciding what to use?
    - How do you decide how many to use?

© 2023 Lawrence EN et al. *JAMA Network Open.*

Next we will shift focus to discussion of why these resources are used, their merits, their drawbacks, and possibly why one would choose not to use them.

- What do these resources add to your learning?
- What do these resources offer that your home institution's curriculum does not?
- Are there times you use a resource more or less?

Thanks, that is very helpful. Now let's shift gears a bit. Comparing your list to the group list, are there resources you were previously aware of but have not used? What are your reasons for non-use of some of these resources? Are there any drawbacks to their use?
Let's discuss how do you decide which resources to use and when.

- Has that changed since you started medical school? Why or why not?
- Is your choice impacted by the course you are taking?
- If you rely heavily on third party resources, what concerns, if any, do you have about using them?
    - (Can prompt): Are there any concerns about missing something in your home institution curriculum or passing your exams? [Allow time for free discussion as above].
    - What are your thoughts about the accuracy of the information in the resources? Can probe: Are you worried about any of the information being incorrect? [Allow time for free discussion as above].

- What advice would you give to incoming M1s about resources? Would you use third-party resources the same way or differently if you did it again?

Thank you for sharing your rationale. We are getting ready to wrap up our focus group. To make sure we don't miss anything that might be on your mind, let's finish with an open-ended question:

- What do you think medical schools should know about third party resources?

Again, thank you for your participation. We will be sharing the information gathered from this focus group in a de-identified fashion with your schools, as well submitting our larger study for publication. Please reach out if you have any feedback, questions, comments, or concerns.

© 2023 Lawrence EN et al. *JAMA Network Open.*
