# Supplementary material for: Preclerkship Medical Students’ Use of Third-Party Learning Resources
Source: JAMA Netw Open. 2023 Dec 4;6(12):e2345971. doi: 10.1001/jamanetworkopen.2023.45971 (PMC10696480; doi:10.1001/jamanetworkopen.2023.45971)
Supplement: Supplement 2. — Data Sharing Statement [file jamanetwopen-e2345971-s002.pdf]

# Data Sharing Statement

Lawrence. Preclerkship Medical Students' Use of Third-Party Learning Resources. *JAMA Netw Open*. Published December 04, 2023. doi:10.1001/jamanetworkopen.2023.45971

**Data**
**Data available:** Yes
**Data types:** Deidentified participant data
**How to access data:** emilycorazon@gmail.com
**When available:** With publication

**Supporting Documents**
**Document types:** Statistical/analytic code
**How to access documents:** emilycorazon@gmail.com
**When available:** With publication

**Additional Information**
**Who can access the data:** Anyone requesting the data
**Types of analyses:** For any purpose
**Mechanisms of data availability:** With investigator support
